# Using team-based learning to optimize undergraduate family medicine clerkship training: mixed methods study

**DOI:** 10.1186/s12909-023-04240-1

**Published:** 2023-06-08

**Authors:** Lisa Jackson, Farah Otaki

**Affiliations:** 1 College of Health Medicine and Life Sciences (CHMLS), Brunel Medical School, London, UK; 2 Strategy and Institutional Excellence, Mohammed Bin Rashid University of Medicine and Health Sciences, Dubai, United Arab Emirates

**Keywords:** Team-based learning, Family medicine, Medical education, Students, Satisfaction, Adult learning, Experiential education, Constructivist learning theory, Social constructionism, Situated learning theory, Team cohesion, Students, Engagement

## Abstract

**Background:**

Team-Based Learning (TBL) is an established educational strategy which has become increasingly popular in the training of healthcare professionals. TBL is highly suitable for teaching Family Medicine (FM) especially that teamwork and collaborative care, in this medical discipline, are at the core of safe and effective practice. Despite the established suitability of TBL for teaching FM, there are no empirical studies that capture the students’ perception of a TBL in FM undergraduate learning experience in the Middle East and North Africa region (MENA).

**Objective:**

The overall objective of this study was to investigate the perception of students regarding a TBL in FM intervention (in Dubai, United Arab Emirates), that was designed and implemented in alignment with a constructivist learning theory.

**Methods:**

A convergent mixed methods study design was utilized to develop a thorough understanding of the students’ perceptions. Qualitative and quantitative data were concurrently collected and independently analyzed. The output of thematic analysis was systematically merged with the quantitative descriptive and inferential findings using the iterative joint display process.

**Results:**

The qualitative findings shed light on the students’ perception of TBL in FM, and the interplay between team cohesion and engagement with the course. As for the quantitative findings, they showed that the percentage of the total average of the Satisfaction with TBL in FM score was 88.80%. As for change in impression of FM discipline, the percentage of the total average was 83.10%. The perception of team cohesion, with a mean of agreement of 8.62(1.34), seemed to be significantly associated with the students’ perception of the team test phase component, only (*P* < 0.05). As for the perception of the level of engagement with the course, with a mean of agreement of 9.29(0.84), it turned out to be significantly associated with the change in impression of FM discipline (*P* < 0.05). Lastly, the joint display analysis showed how the quantitative and qualitative findings built upon each other, revealing how best to leverage TBL in FM trainings.

**Conclusion:**

The current study showed that TBL embedded in a FM clinical clerkship was well-received by students. It is worth leveraging the lessons learned from the first-hand experience reported upon in the current study to optimize the utilization of TBL in FM.

**Supplementary Information:**

The online version contains supplementary material available at 10.1186/s12909-023-04240-1.

## Introduction

Team-Based Learning (TBL) is an established educational strategy which has become increasingly popular in the training of healthcare professionals [1]. TBL has a clear structure which can be applied across many subjects, allowing for the analysis and resolution of complex problems. The role of the instructor changes to that of a facilitator who provides guidance and feedback, while the learner is placed in the center as an active participant [2]. It is well recognized that TBL fits into the realm of constructivist experiential learning theory by assuming that the learners are activating previous learnings, while undergoing processes of assimilation and accommodation of new information [3]. As such, TBL, from the perspective of social constructionism, becomes a process of active adaption, where a small group of students are collectively learning through their social interactions [4]. As such, the analytic focus shifts from the “individual as a learner” to “learning as participation in the social world”.

Similar to the case with any practice-based learning, crafting TBL learning experiences requires considering the individuals, their experiences, and the overall learning environment [5]. The underlying premise is that the learners are self-directed and self-regulated, are intrinsically motivated to learn, and tend to exercise analogical reasoning [6]. Also, it is assumed that the adult learners have previous knowledge and experience, that take the form of a malleable resource for learning. Within this context, the adult learners can be considered to have mental models that guide their attitudes and behaviors. It is worth complementing this understanding of adult learning with Kolb’s experiential learning virtuous cycle which suggests for the TBL experience to start with hands-on learning experiences in safe environments, followed by guided reflections with skilled mentors, then abstract conceptualization where the students adapt their mental models, and finally active experimentation where the students test their modified mental models [7]. Anchoring all of this in social constructionism enables fostering the learning that occurs through the embeddedness of the learners within the environment and the social interactions of which they are a part [4, 8].

Many benefits of TBL have been previously reported upon [9]. TBL holds the potential to provide an enriching and rewarding learning environment that motivates students to build on their basic knowledge and put into practice what they learn [10]. TBL enables student-centered, active learning experience [11] and enhances student engagement and satisfaction, the quality of the student learning experience, and the student performance [12]. A previously conducted study in Lebanon revealed that TBL is effective in teaching critical appraisal to preclinical medical students [13]. In the context of a pediatric clerkship, students not only favored TBL over lectures, but also performed better in corresponding assessments. TBL clearly increases appreciation for team work [14]. A comparison of TBL with Lecture-based Learning (LBL), in Indonesia, showed that TBL had a positive effect on clinical reasoning skills [15]. Furthermore, incorporating TBL into the delivery of medical programs is believed to assist in preparing medical students for the demands of increasingly complex health systems [10].

TBL is highly suitable for teaching Family Medicine (FM) for many reasons [16, 17] especially that teamwork and collaborative care, in this discipline, are at the core of safe and effective practice [18]. The clinical learning environment tends to be treated by FM practitioners as a community-of-practice for students and residents alike. Accordingly, TBL can enable the creation of a mini environment that replicates the clinical setting. It also offers the opportunity to integrate continuous assessment which is known to maximize the development of professional knowledge, skills, and behaviours, even from the earliest stages of undergraduate medical training [19]. TBL also helps in developing an understanding of communities and in increasing responsiveness to their needs [20]. Despite the suitability of TBL for teaching FM, there is limited evidence of deploying this teaching methodology in undergraduate FM clerkships. As such, the true potential of TBL is perhaps still not leveraged in this area.

To the best of the authors knowledge, there are no empirical studies that capture the students’ perception of a TBL in FM undergraduate learning experience in the Middle East and North Africa region (MENA). Therefore, the overall objective of this study was to investigate the perception of students regarding a TBL in FM intervention [21, 22], that was designed and implemented in alignment with a constructivist learning theory (Situated Learning Theory- SLT). In addition, the study aims at uncovering the factors, related to the TBL in FM experience, which affected the students’ extent of satisfaction with the educational intervention. Accordingly, this study addresses the following research questions:

1. How did the students perceive the TBL in FM experience?
2. How satisfied were the students with the TBL in FM experience, and what variables were associated with the students’ extent of satisfaction?
3. How best to leverage TBL in FM training?

## Methods

### Context of the study

Mohammed Bin Rashid University of Medicine and Health Sciences (MBRU) opened its doors in academic year 2016–2017 in Dubai, in partnership with Queen’s University Belfast (QUB), UK. Among the University’s offerings is the Bachelor of Medicine, Bachelor of Surgery program (MBBS). The academic year 2019–2020 marked the first round of implementation of the clinical phase of the MBBS with FM embedded in equal proportion with each of the other specialities: Internal Medicine, Surgery, Paediatrics, and Psychiatry. The introduction to clinical practice during the pre-clinical phases heavily relied on contact with simulated patients for developing skills in both history-taking and clinical examination. Most theory was taught using teacher-centric, lecture-based didactic sessions. Students in the first cohort of the MBBS at MBRU came from 17 countries, including most Gulf States, and from further afield including the Indian Sub-Continent, Africa, Europe, and North America. They were the first cohort to graduate with MBBS towards the end of the academic year 2021–2022. The gender distribution is skewed towards female (70%), and 19% of the cohort identify as local (versus 81% as expats or international).

In Dubai, there is a range of private providers as well as a public service regulated by the Dubai Health Authority [23]; FM is available in both sectors. The Government of Dubai sees primary care as a major policy priority with the development of further public provision planned in the coming years. The Dubai Health Authority FM residency program has been sufficiently successful that it is possible to have a fully Emirates-trained specialists’ workforce [24]. Further, in 2021, it was announced that there is a goal to develop a full Academic Health System, in which primary care is likely to play a large role [25]. The establishment of a new Medical School and of an Academic Health System is in alignment with the longer-term goals of the Government of Dubai of improving public health, and increasing the number of Emiratis who are locally trained and who could go on to work locally in a wide range of specialties.

### Description of the TBL in FM clerkship

**Fig. 1:**
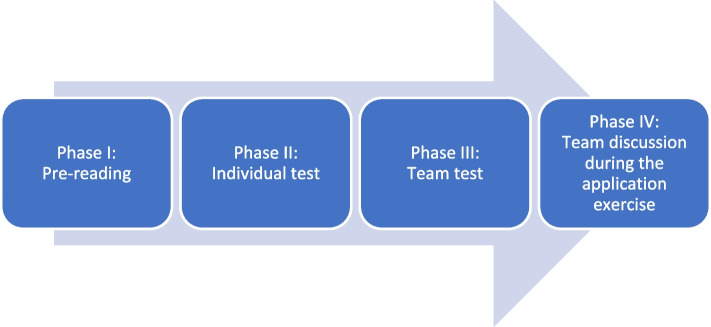
Four phases of TBL process

A series of seven four-hour classes of clinical FM curriculum was developed and implemented by LJ using four phases of the TBL methodology: Pre-reading, Individual test, Team test, and Team discussion (Fig. 1), for the year 4 cohort of 45 students, taught from August 2019 until July 2020. The series of 7 classes was repeated 5 times, once for each of the 5 FM rounds taking place during the academic year 2019–2020.

The TBL process included the Readiness Assurance Process (RAP) [i.e., Individual Readiness Assurance Test (IRAT) and Team Readiness Assurance Test (TRAT)], developing application cases, and recruiting subject matter experts (drawn from Adjunct Faculty) to attend, expand, and/ or clarify concepts [26]. Pre-reading consisted of two case-based FM exercises from the American Aquifer series [27] which focus on topics that are common in FM (e.g., Well Person checks, fatigue, unwell children, and headaches). Seven application cases were designed for the purpose of this TBL to develop among the students the ‘Family Doctor’ thinking process, which enables seeing undifferentiated cases. These application cases were designed using the ‘Significant Problem, Same Problem, Specific Choice, and Simultaneous Report’ (4S) technique [22]. However, each new application case (in contrast to the pre-reading cases) was designed in collaboration with local doctors who were able to check for local relevance and authenticity, to make sure that (in alignment with SLT), the various population groups and types of presentations seen in Dubai were effectively represented in this TBL, while maintaining its wider applicability. Selected questions highlighted specific skills for focus such as communication skills with individuals facing language barriers, or dealing with stresses encountered by migrant or expatriate workers. To foster the adult learning that is core to the constructivist learning theory, the cases were characterized by a range of correct answers (as opposed to a Single Best Answer- SBA), leading to several reasonable approaches to dealing with any specific case which is key to the targeted ‘Family Doctor’ thinking process. Students were expected to justify their clinical management approaches, making sure to deal with urgent acute issues first, and then defining how they would deal with more long-term, chronic problems (and/ or problems-in-evolution which may require further investigation and appropriate management). The idea was to simulate real practice, which the students could then compare with their clinical experiences in the outpatient and hospital settings. Then gradually, over the seven-week period, they get to improve their skills at analyzing undifferentiated cases, while working in teams, mimicking situated multidisciplinary working. This was possible because students were in different placements, and were gradually building their knowledge and skills in a real clinical setting, then checking their knowledge and practicing these skills in the TRAT and application case work. During teaching sessions at the University, subject matter experts (i.e., select adjunct faculty) were invited to expand on generic topics such as dealing with uncertainty, or more specific topics, such as: immunization and screening both internationally and within the local context (referring to local and international guidelines, or consensus-based best practice).

Students received a small grade (2.5% of final end-of-year mark) for both IRAT and TRAT performances combined. Teams were formed at the start of the round by doing an icebreaker to discover key characteristics of the individuals which enabled the formation of balanced, heterogenous teams. Feedback after the TRAT was immediate, with elaboration on correct answers, and discussions on why some answers were incorrect, revealing any misconceptions or misunderstandings. Burning questions were solicited and appeals were allowed. During the application cases, team discussions were facilitated, with the select adjunct faculty instructed beforehand not to ‘teach’ but to ask questions which encouraged students to find the answers themselves. Since the groups were small (5–6 members) and there were only two groups in each round, simultaneous presentation of answers was straight-forward. Students were enabled to see both teams’ answers side-by-side initially using flipcharts and later a Gallery Walk function in computer software. Peer feedback was also included (but not appraised in this study). The COVID-19 pandemic affected, from March 2020 through July 2020, the intervention under investigation. This led to the rapid transition of the face-to-face classroom to distance learning for the last one and half rounds (total 12 weeks out of 40) [26, 28, 29].

### Research design

**Fig. 2:**
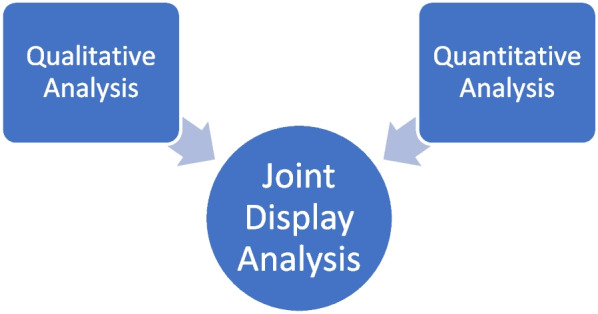
Convergent mixed methods study design (showing how the first layer of analysis through the iterative join display analysis process leads to meta-inferences)

A convergent mixed methods study design [30], which has been frequently relied on in health professions’ education research [28, 31, 32], was utilized to develop a thorough, systemic understanding of the students’ perceptions regarding the TBL in FM experience (Fig. 2). This study is characterized by three phases. In the first phase, qualitative and quantitative data were concurrently collected. The qualitative and the quantitative data were analyzed each independently in the second phase of the study. In the third phase, the output of qualitative analysis was merged with the quantitative analysis using the iterative joint display analysis process [33, 34]. The integration of data types (i.e., qualitative and quantitative) is meant to raise the validity of the generated findings. Ethical approval for the study was granted by the Mohammed Bin Rashid University of Medicine and Health Sciences Institutional Review Board (Reference # MBRU-IRB-2019–015).

### Data collection

The data was collected using a survey that aimed at evaluating the extent of satisfaction with the TBL in FM experience, and perceived team cohesion and level of engagement with the course throughout the experience, as well as the change in the students’ impression towards the discipline of FM. The survey was composed of three parts (Appendix 1: Data Collection Tool). It was developed taking into consideration the need to efficiently obtain evaluation data so as not to burden the students, and to solicit for both quantitative and qualitative feedback. Moreover, it was based on a mix of brief narrative questions similar to Stead [35] and elements similar to existing TBL evaluation questionnaires such as that developed by Parmelee [22] (Appendix 2: Detailed Description of Data Collection Tool).

The survey was administered five times, once to each of the FM clerkship groups, towards the end of the corresponding round. The data collection was carried out by FO who was independent of the teaching and assessment of the TBL in FM program. Students provided informed consent for participation. An information page appeared when the participating students clicked on the survey link (before they started the survey) that indicated to them that their participation is completely voluntary, and that their privacy and data confidentiality would be protected. No personal identifiers were recorded. Each participant was serially assigned a unique identifying number (1 through 45), preceded by the number of the round (1 through 5). For example, the 10^th^ student who is from the 2^nd^ round of the clerkship is identified as “2–10”.

### Data analysis

#### Qualitative analysis

The qualitative data were inductively analyzed, by two researchers (LJ and FO), using the Braun and Clarke (2006) six-step framework [36] recently endorsed in health professions’ educations [37–40]. As such, all the qualitative data collected using the tailored study survey got retrieved and systematically analyzed. The two researchers started by familiarizing themselves with the extracted data. Following that, the researchers generated initial codes to fragments of the data that resonate the most with the research questions. After attaining data saturation where no new codes were arising from the analysis, the researchers moved to the third step of the analysis process where they thoroughly reflected on the generated codes and all the ways in which they could relate to one another to cluster the codes into themes. Any disagreement between the two researchers was perceived as an opportunity to reinforce the common ground between them, which is favored in qualitative research [41], where, at any discordance, the discussion was pursued until a consensus was attained. These themes were further investigated and in turn refined as part of the fourth step of the analysis. Then, as part of the fifth step, a code for each overarching theme was pinpointed and defined. The last step constituted reflecting upon the output of analysis by narratively reporting upon the results, in alignment with established guidelines [41–43].

#### Quantitative analyses

The quantitative data were analyzed using SPSS for Windows version 27. The descriptive analysis consisted of computing an overall satisfaction score of the five components of the Satisfaction with TBL in FM tool. The mean and standard deviation for this overall satisfaction score and each component of it, and that of the perceived team cohesion and level of engagement with the course, and of the change of impression of the FM discipline were calculated. The validity tests of Cronbach’s Alpha, and the Principal Component Analysis (PCA), accompanied by bivariate analysis, were performed to assure the internal consistency and external variance, respectively, of the Satisfaction with TBL in FM tool.

To select the appropriate inferential analysis tests, a test of normality was conducted for each of the five components of the Satisfaction with TBL in FM tool and its overall score, and values of perceived team cohesion and level of engagement with the course, and change in impression of FM discipline. The data were all found to be not normally distributed. Accordingly, Kruskal–Wallis one-way analysis of variance test was used to assess the potentiality of associations between the following variables: the Satisfaction with TBL in FM, perceived team cohesion, perceived level of engagement with the course, and change in impression of FM discipline.

#### Data integration/ joint display analysis process

After the completion of the independent data analysis of quantitative and qualitative data, the generated findings were integrated using the joint display analysis process [44]. This stage enabled drawing meta-inferences from the mapping of findings generated from each of the independent preceding analyses (Fig. 2) [45]. As such, the researchers were able to identify where the findings build upon (or at least confirm) each other. This systemic technique also allowed for identifying findings that contradict each other.

## Results

### Qualitative data

The qualitative findings can be grouped into two topics: the students’ perception of TBL in FM (with detailed insight into each phase of the learning experience), and the interplay between team cohesion and the engagement with the course (and how the heightening of either of those aspects, or both together, maximizes the benefits of a TBL in FM experience such as the one under investigation).

### Students’ perception of TBL in FM

**Fig. 3:**
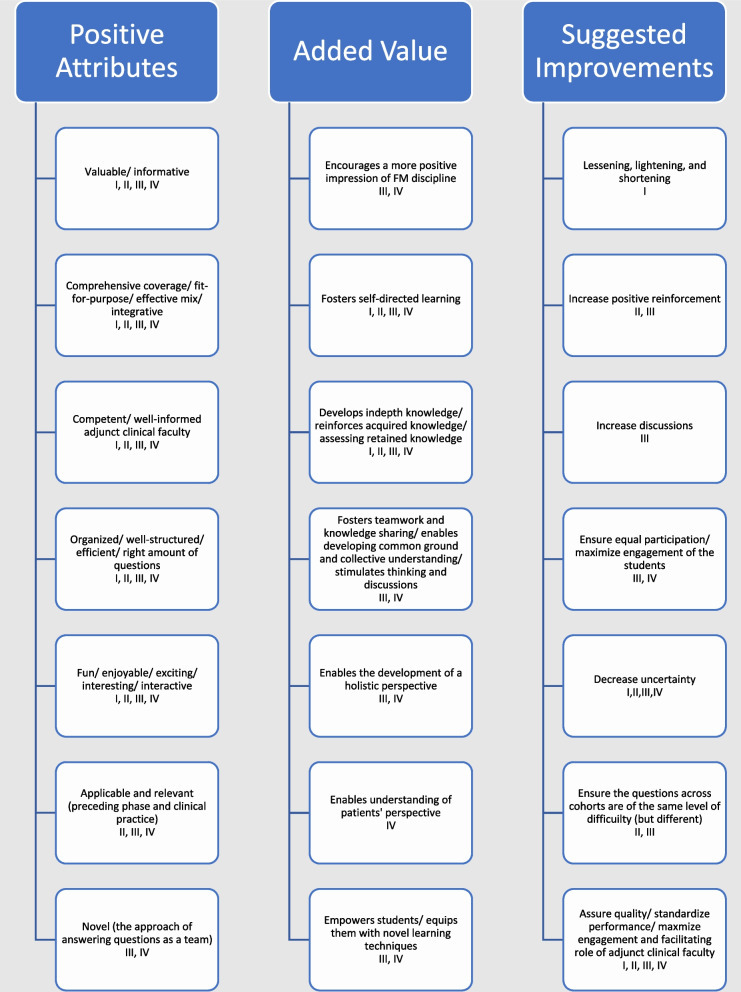
Study’s conceptual framework: 7X3 Students; perception of TBL in FM [roman numerals refer to the TBL phase(s) in which the respective category code surfaced]

The output of analysis of the qualitative data related to the TBL phases was fitted into three interrelated themes, namely: positive attributes, added value, and suggested improvements (Fig. 3, and Appendix 3: Narrative Reporting on Output of Qualitative Data around Students’ Perception of TBL in FM).

### Team cohesion and engagement with the course

**Fig. 4:**
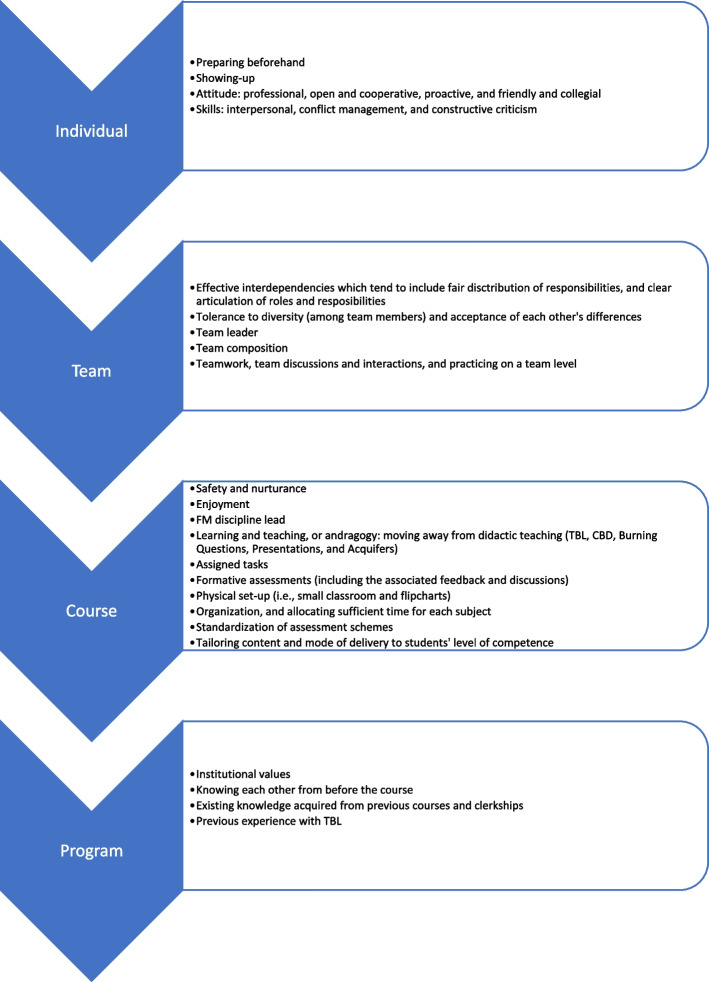
Facilitators/ moderators of the relationship between team cohesion and engagement with the course, across differing levels of analysis

The qualitative data analysis also revealed how the levels of team cohesion and of engagement with the course appear to positively reinforce each other. The students identified a set of facilitators/ moderators that appear to be fueling this mutually-reinforcing relationship. These facilitators can be divided into four levels of analysis: individual, team, course, and program (Fig. 4).

The students also pinpointed benefits of fostering of both of those aspects of the experience (i.e., team cohesion and engagement with the course), which include:

- Enhancing critical thinking
- Excercising clinical correlation
- Widening horizons and developing a holistic view
- Seeing perspectives of other team members and becoming more empathetic
- Promoting self-directed learning and self-monitoring, and identifying personal development needs
- Bonding with each other and spending time together
- Learning from each other, and exchanging information and knowledge
- Enhancing understanding of the subject matter and preparing for assessments
- Enhancing teamwork skills, reaching consensus, and increasing cooperation

### Quantitative analyses

Out of those 47 students, as per Table 1, 45 responded (i.e., overall response rate = 95.74%).

**Table 1:** Response rates across rounds

Clerkship round	Number of responses	Total number of students	Response percentage
1	9	9	100
2	9	10	90
3	9	9	100
4	9	9	100
5	9	10	90
Total	45	47	95.74

The reliability score of Cronbach’s Alpha for the Satisfaction with TBL in FM tool was 77.60%. The percentage of the total average of the Satisfaction with TBL in FM score was 88.80%, as per Table 2.

**Table 2:** Output of descriptive quantitative analysis

Variable		Mean (SD)	Percentage of the mean	Category
Satisfaction with TBL in FM	Component 1: Pre-reading phase	8.36 (1.33)	83.60%	A-SA
	Component 2: Individual test phase	8.96 (1.11)	89.60%	A-SA
	Component 3: Team test phase	9.33 (1.09)	93.30%	A-SA
	Component 4: Team discussion phase	8.80 (1.36)	88.00%	A-SA
	Component 5: Overall Satisfaction with TBL in FM	8.96 (1.11)	89.60%	A-SA
	Satisfaction with TBL in FM score	44.40 (4.33)	88.80%	A-SA
Team cohesion		8.62 (1.34)	86.20%	A-SA
Level of engagement with the course		9.29 (0.84)	92.90%	A-SA
Change in impression of FM discipline		8.31 (1.77)	83.10%	A-SA

*A* Agree and *SA* Strongly Agree

According to the PCA (Kaiser–Meyer–Olkin Measure of Sampling Adequacy), 77.70% of the variance can be explained by the instrument (*p* < 0.001). Along the same lines, the Bivariate Spearman Correlations showed how the changes in the Satisfaction with TBL in FM score can be explained by changes in all five components. Moreover, Components 1 and 4 were not associated with each other (*p* > 0.05), although each was associated with Components 2, 3, and 5, independently. Components 2, 3, & 5 were each associated with all the rest of the components of the tool (*p* < 0.05).

As for the perception of team cohesion and of the level of engagement with the course, and change in impression of FM discipline, the percentages of the total average were 86.20% and 92.90%, and 83.10%, respectively, as per Table 2.

The perception of team cohesion, with a mean of agreement of 8.62(1.34), seemed to be significantly associated with the perception of the team test phase component, only (*P* < 0.05), and not associated with the rest of the variables under investigation: perceptions of the rest of the components of the Satisfaction with TBL in FM tool (i.e., pre-reading, individual test, team discussion, and overall satisfaction) and its overall score, and level of engagement and change in impression of FM discipline (P > 0.05). As for the perception of level of engagement with the course, with a mean of agreement of 9.29(0.84), it turned out to be significantly associated with the overall Satisfaction with TBL in FM score and all its five components, and perception of change in impression of FM discipline (*P* < 0.05).

### Joint display analysis

The joint display analysis (Table 3) showed how the qualitative and quantitative data built upon each other, resulting in second-level inferences (involving three parameters). In relation to the success factors of the TBL in FM experience, the quantitative data showed that it is essential for the students to be satisfied with each of the TBL phases, independently, to be satisfied with the experience, as a whole. It also highlighted the importance of the test phases. For the same parameter (i.e., success factors of the TBL in FM experience), the qualitative data pinpointed the actual positive attributes of the experience as perceived by the students.

**Table 3:** The study’s joint display showing how the data is effectively integrated to create a whole that is more than the sum of its parts

Qualitative →	Meta-inferences parameters	← Quantitative
Perceived positive attributes	**1. Success factors of TBL in FM experience**	• Each of the four components need to be considered satisfactory for the experience, as a whole, to be considered satisfactory (by the students) • Satisfaction with pre-reading phase (i.e., 1) is not associated with the satisfaction with the team discussion phase (i.e., 4) • Perception of pre-reading and team discussion phases (i.e., 1 & 4), independently, are not associated with the overall level of students’ satisfaction • The test phases (i.e., 2 & 3) can be considered “foundational”: satisfaction with each of these phases, independently, is associated with satisfaction with each of the rest of the TBL in FM phases, independently (and with the overall level of students’ satisfaction with the TBL in FM)
• Perceived positive attributes • Perceived added value • Suggested improvements	**2. Perceived efficacy of TBL in FM experience**	• Each phase was perceived as satisfactory • The experience, as a whole, was perceived as satisfactory • Positive change in FM impression due to the TBL in FM experience was evident • Positive change in FM impression was associated with overall level of students’ satisfaction with the TBL in FM
• Mutually reinforcing relationship between team cohesion and level of engagement with course • A set of “facilitators” seem to be fuelling the abovementioned mutually- reinforcing relationship	**3. Variables associated with perceived efficacy of TBL in FM experience**	• Team cohesion was associated with increased satisfaction with phase 3: team test phase • Level of engagement with the course was associated with satisfaction with the intervention (as a whole, and each phase of it, independently) • Level of engagement with the course was associated with positive change in FM impression

The second parameter, namely: the perceived efficacy of the TBL in FM experience constituted the core of the joint display model, where the quantitative analysis showed that the experience in its entirety was satisfactory for the students, along with each of its phases, independently. The quantitative data also showed how the TBL in FM experience enhanced the students’ impression of the discipline of FM, where this variable may be playing a moderating role between exposing the students to the intervention with their overall level of satisfaction with the entailed learning experience. The qualitative analysis, in relation to the second parameter of the joint display (i.e., the perceived efficacy of the TBL in FM experience), showed several other benefits of the intervention, in addition to enhancing students’ impression of FM discipline.

In relation to the third parameter of the current study’s joint display (i.e., variables associated with perceived efficacy of TBL in FM experience), team cohesion and level of engagement with the course seem to have played a fundamental role in the TBL in FM experience, where the qualitative data analysis showed a mutually reinforcing relationship between those variables, and the facilitators that augment the interplay between those two variables.

## Discussion

In the context of this study, the TBL in FM learning experience was well-received by the students. They pinpointed the positive aspects of the experience that enabled them to thrive, how this teaching modality added value to the learning experience, and the opportunities available to improve the application of TBL to a FM clerkship. The output of the educational intervention, and the mixed methods research work reported upon in the current study, revealed that the ‘core’ of the TBL is the RAP (i.e., IRAT and TRAT); this was derived from the observation that the perception of the test phases (i.e., 2 & 3) were clearly associated with the perception of the overall TBL process, where the students considered the intervention to be satisfactory. This reinforces the importance of assuring the quality of the questions, particularly when they are used for summative assessments. It may also be an indication of a need to revisit the design and implementation of the application exercises.

The current study’s results also imply that there is value in emphasizing upfront the importance of the RAP phases when setting the stage for the learning experience. A previously conducted study suggests that students who do not consistently prepare for TBL, as reflected by low IRAT scores, exhibit poorer performance on the final examination. The lack of preparation, among the students, is likely to affect the efficacy of this learning method [46]. One study also highlighted the potential usefulness of monitoring IRAT scores for early identification of struggling students who may need additional support [47]. In terms of psychological theory, it has been suggested that the preparatory phase followed by IRAT taken after a gap (such as a night’s sleep) may contribute to the brain’s capacity to consolidate information by facilitating retrieval soon after it is learned. The TRAT group discussion helps students in turn activate their knowledge, help one another, and reinforce that knowledge. In so doing, the students get to nurture their relationships that make the class a safe place to learn [48]. This is also demonstrable in the fact that TRAT scores are invariably higher than the IRAT [49].

It is established that supporting the students in developing strong background knowledge enables them to discuss cases comprehensively with peers which in turn sharpens their clinical reasoning [15]. Although the qualitative data, in the current study, proved that the preparation phase was crucial for the learning experience, the students’ perception of the pre-reading and of the application case, independently, were not associated with the overall level of students’ satisfaction (according to the quantitative data). In other words, although the students were aware of the significance of preparing for the assessments, the nature of the preparatory activity integral to this phase might have been perceived as burdening. In fact, the qualitative data also showed that the students specified lessening the reading load as an opportunity to improve the learning experience. Accordingly, when selecting reading materials, it is important to remain mindful of the overall studying load, and perhaps differentiate, in communicating to the students, between the mandatory reads and the optional ones.

Students are of course influenced by numerous factors, leading to variations in focus. For example, the implementation of TBL in a Middle Eastern pre-clinical course was seen as contributing to the students’ sense of responsibility, but students also appeared to score lower in the IRAT during weeks when other summative exams were taking place [50]. This highlights the need to always consider the overall timetabling of the program. There has been some discussion by Burgess et al. [51] about the best placement of TBL within a curriculum. It was suggested that TBL is particularly effective for building basic science knowledge, and case-based learning promotes the development of further clinical reasoning skills. The current study’s finding of relative disconnect between the pre-reading and the application cases arose (most likely) from the fact that our application cases were designed to extend students’ knowledge beyond the prior reading into the domain of more complex problem-solving including clinical reasoning. The RAP was clearly most useful for checking engagement with the pre-reading and ensuring a suitable level of baseline knowledge to tackle any one case.

A specific variable which seemed to influence perception of the TRAT, among the study’s participants, was the perceived team cohesion. There are several factors which can influence this, notably team size, and length of time working together. The teams in the current study consisted of 5–6 students, a number often quoted as optimal based on the presumption that teams need to be large enough to have sufficient collective knowledge and skills [52]. In the Thompson et al. [52] study, which looked at teams of 5–7 members, individuals from larger teams performed better at the end of the course on the National Board of Medical Examiners (NBME) knowledge test of psychiatry. In the intervention of the current study, students also worked together for a total of 28 h in the classroom over 7 weeks, which may have enabled gradual team building along with the gradual building of problem-solving skills in the FM-based problems. This is consistent with suggestions in the literature that state that sufficient time is needed to maximize the benefits of TBL [53]. In terms of facilitation, this finding also points to the importance of team formation and maintenance exercises. The former happens prior to the experience. The latter requires intentional intervention throughout the phases. However, there is also more recent evidence that suggest that the mode of initial heterogenous team formation may not make any difference to student perception of the experience or the results of the TRAT [54].

The students’ engagement with the FM course, in the current study, appeared to be significantly associated with their level of satisfaction with the TBL phases, independently and collectively. This sheds light on the necessary set of skills of the TBL lead facilitator, who needs to think systemically, while regularly zooming into the intricacies of the learning and teaching experience to assure the quality of the delivery of each phase. This highlights the importance of training for sustainability and working towards attaining the long term ‘buy-in’ of all involved faculty and external subject matter experts [55].

Students, in the current study, did not appear to be put-off by the summative grading of the RAP (i.e., IRAT and TRAT), and indeed the comments suggest that the students’ engagement improved because of implemented grading scheme as it incentivized pre-reading and was perceived as a low-stakes testing. One study in Singapore supports this observation in respect to the IRAT [56]. Results of the respective study suggest that grading had a positive effect on students’ performance. When the students’ performance was graded, they appeared to engage more with the content of the TBL sessions, namely: the pre-class materials, and in turn to perform significantly better on IRAT. This ultimately improved their performance on their examination scores. Accordingly, it may be useful for the IRAT, at least, to continue to be used as a summative assessment. Carrasco, Behling, and Lopez [57] suggested that modifications in TBL incentive structure may provide more tangible rewards for pre-class preparation, especially for students struggling to keep-up with the delivered content.

By coupling an ongoing FM clerkship of over 8 weeks with a series of TBL sessions, students can apply their experiences in clinics to all aspects of the TBL process. As they learn more in clinics about how FM doctors actually go about their daily activities, the students can perform increasingly well in the application exercises which involve complex problem solving. They begin to see that the cases are ‘real’, and therefore, matter to their learning. A recent review in 2020 [58] indicated that TBL is being increasingly used in medical curricula, but still predominantly in pre-clinical phases. The design of the clinical program under investigation allowed for easy implementation of TBL with a once weekly four-hour slot for teaching in any format the discipline lead chose. In practice, flipped classrooms made much more sense than a four-hour continuous lecture series each week, or even problem-based learning which normally would require meeting twice weekly. The value of such a hybrid model is reflected upon in the literature, where for example combining TBL with Lecture Based Learning (LBL) appeared acceptable to the students and resulted in better outcomes than either method alone in neurology clerkships [59]. Such models are believed to enable students to absorb a large amount of abstract and complex course materials in a short period [58].

A key element of the TBL in FM implemented at MBRU was the involvement of multiple adjunct faculty who were, through their engagement, simultaneously getting trained in facilitation skills along with acting as content experts. The MBRU discipline lead (LJ) was present in the group, on a weekly basis, assuring the quality of the TBL process and the content covered, and ensuring that the content experts avoided lecturing. It was later possible to hand over this supervisory role to those newly trained facilitators who gained confidence in handling this responsibility, enabling new subject experts to join the process as it was unfolding. This may have contributed to some of the positive perception of the program because there was continuity in the form of one regular facilitator, and content experts were well supported on a week-to-week basis. This kept the process controlled and consistent from one round to the next, and students knew what to expect. Another previously conducted study revealed that the involvement of experienced, senior clinicians as facilitators, sharing their expertise within a clinical context, elicited effective student engagement in their own learning [10]. Moreover, the involvement of two teachers as a facilitator and a content expert respectively has been investigated in another study in relation to how those teachers can use their skills in complementary ways [60]. The analysis showed that trained facilitators are much better at using open questions, while content experts are more inclined to give information. Another benefit of a regular presence in the classroom is the development of familiarity with equipment and/ or computer software, troubleshooting problems, and keeping the class going when technical glitches occur. Co-teaching also built relationships with the clerkship locations, as many of the doctors attending the sessions also taught the students in their clinics. The discipline lead (LJ) built relationships with these doctors, role modelling teamwork.

During the problem solving of the application cases, although students had to make a specific choice and in turn justify this choice, there were several possible correct answers to any of the application cases. This was intended to ensure a clearer representation of ‘real life’ FM problems where there may be multiple problems to manage, and several reasonable avenues. The key skill for the students to develop was the ability to recognize what was urgent, and what conditions required prevention/ future screening and what conditions needed long-term management. Students also had to discuss differential diagnoses, clinical reasoning, management approaches for specific conditions, and communication skills required for each case including incorporation of role plays. Facilitators highlighted the ‘ideal’ approaches during the simultaneous reporting phase after hearing justifications for the approaches rather than assessing a multiple-choice question. There is support for this approach for healthcare-based application cases [61]. Some of this evidence considers that the demand for a (very) specific choice in healthcare TBL may reduce the discussion and oversimplify situations [62], and it has been argued in response that teachers may want to avoid the writing of further multiple-choice questions for application case answers [63]. It could be argued that there is an equal work demanded in creating a model answer or in taking on board in real time the reasonable arguments of senior students in a complex case.

In the TBL in FM implemented at MBRU there was the advantage of having to deal with only 2 teams per round which allowed for deeper discussions. For larger groups in TBL, using more free text responses is harder to implement, as the process of working through many long documents is less practical. The downside, as pointed out in the TBL literature, is that not adhering to the SBA format can hamper evaluation and comparison of different studies in the area. This would be an important subject for further discussion and research [64].

This study is characterized by a set of limitations. Although the choice of mixed methods study design offered plenty of in-depth insights, the generalizability of the findings is limited to schools that are contextually similar to MBRU. The onset of the pandemic, amid the investigation, made the context of the current study even more unique. Hence, it is recommended for future studies to investigate the application of TBL in FM across a combination of differing contexts. Such a study can be longitudinal in nature to allow for establishing causality in between variables since this study was restricted to uncovering associations. Moreover, despite all the efforts directed towards maintaining consistency, as part of assuring the quality of the experience, inevitable variation likely occurred across the rounds. For example, in terms of exposure (clinical or otherwise), students had differing experiences when starting their TBL in FM, from no prior clinical experience in the first round, to 32 weeks of learning in 4 other disciplines (Psychiatry, Internal Medicine, Pediatrics, and Surgery) by the last round. This variation might have affected the participants’ perception of the experience. It is worth undertaking a similar exploration around a clinical placement that is not rotation-based.

## Conclusion

The current study, which describes the implementation and evaluation of a TBL program embedded in a FM clinical clerkship rotation, showed that this approach was well-received by students. It is worth leveraging the lessons learned from the first-hand experience reported upon in the current study to optimize the utilization of TBL in FM.

## Supplementary Information

**Additional file 1: Appendix 1.** Family Medicine Team Based Learning (Round X) Evaluation.

**Additional file 2: Appendix 2.** Detailed Description of Data Collection Tool.

**Additional file 3: Appendix 3.** Narrative Reporting on Output of Qualitative Data around Students’ Perception of TBL in FM.

## Data Availability

The datasets used and/or analyzed during the current study are available from the corresponding author on reasonable request.

## Abbreviations

TBL: Team-Based Learning
FM: Family Medicine
UK: United Kingdom
UAE: United Arab Emirates
SLT: Situated Learning Theory
MBRU: Mohammed Bin Rashid University of Medicine and Health Sciences
QUB: Queen’s University Belfast
MBBS: Bachelor of Medicine, Bachelor of Surgery
RAP: Readiness Assurance Process
IRAT: Individual Readiness Assurance Test
TRAT: Team Readiness Assurance Test
SBA: Single Best Answer
IRB: Institutional Review Board
PCA: Principal Component Analysis
NBME: National Board of Medical Examiners
