# Supplementary material for: Using team-based learning to optimize undergraduate family medicine clerkship training: mixed methods study
Source: BMC Med Educ. 2023 Jun 8;23:422. doi: 10.1186/s12909-023-04240-1 (PMC10248977; doi:10.1186/s12909-023-04240-1)
Supplement: Supplementary file 1 — Additional file 1: Appendix 1. Family Medicine Team Based Learning (Round X) Evaluation. [file 12909_2023_4240_MOESM1_ESM.docx]

Appendix - Family Medicine Team Based Learning (Round X) Evaluation

Part I- Satisfaction with the TBL in FM experience:

* 1. Perception of the Phase I- Pre-reading Phase:

We would like to know what you think about the preparation or pre-reading that you have been asked to do before each class session.
Please select the rate that seems most appropriate.

| 0 | 1 | 2 | 3 | 4 | 5 | 6 | 7 | 8 | 9 | 10 |
| --- | --- | --- | --- | --- | --- | --- | --- | --- | --- | --- |
| Waste of Time |  |  |  |  |  |  |  |  |  | Superb |

* 2. Please give two reasons for your score:

Insert Comment

* 3. Please suggest two possible improvements to the Pre-reading Arrangements:

Insert Comment

* 4. Perception of the Phase II- Individual Test Phase:

We would like to know what you think about the individual test that you do at the start of each class session.
Please select the rate that seems most appropriate.

| 0 | 1 | 2 | 3 | 4 | 5 | 6 | 7 | 8 | 9 | 10 |
| --- | --- | --- | --- | --- | --- | --- | --- | --- | --- | --- |
| Waste of Time |  |  |  |  |  |  |  |  |  | Superb |

* 5. Please give two reasons for your score:

Insert Comment

* 6. Please suggest two possible improvements to the Individual Test Arrangements:

Insert Comment

* 7. Perception of the Phase III- Team Test Phase:

We would like to know what you think about the team test that you do at the start of each class session.

Please select the rate that seems most appropriate.

| 0 | 1 | 2 | 3 | 4 | 5 | 6 | 7 | 8 | 9 | 10 |
| --- | --- | --- | --- | --- | --- | --- | --- | --- | --- | --- |
| Waste of Time |  |  |  |  |  |  |  |  |  | Superb |

* 8. Please give two reasons for your score:

Insert Comment

* 9. Please suggest two possible improvements to the Team Test Arrangements:

Insert Comment

* 10. Perception of the Phase IV- Team Discussion Phase:

We would like to know what you think about the team discussion exercises that you do during each class session.

Please select the rate that seems most appropriate.

| 0 | 1 | 2 | 3 | 4 | 5 | 6 | 7 | 8 | 9 | 10 |
| --- | --- | --- | --- | --- | --- | --- | --- | --- | --- | --- |
| Waste of Time |  |  |  |  |  |  |  |  |  | Superb |

* 11. Please give two reasons for your score:

Insert Comment

* 12. Please suggest two possible improvements to the Team Discussion Exercises:

Insert Comment

* 13. Perception of the Overall TBL in FM Experience:

We would like to know what you think about the way that classes have been conducted during this semester.

Please select the rate that seems most appropriate.

| 0 | 1 | 2 | 3 | 4 | 5 | 6 | 7 | 8 | 9 | 10 |
| --- | --- | --- | --- | --- | --- | --- | --- | --- | --- | --- |
| Waste of Time |  |  |  |  |  |  |  |  |  | Superb |

* 14. Please give two reasons for your score:

Insert Comment

* 15. Please suggest two possible improvements to the way that these classes have been conducted:

Insert Comment

Part II- Team Cohesion and Level of Engagement:

* 16. Subsection I- Team Cohesion:

We would like your comments on how you experienced "Team Cohesion" in this course, where "Team Cohesion" is defined as the degree to which you formed bonds that help your team become united and 'stick together' in turn facilitated carrying-out your team tasks.

Please select the rate that seems most appropriate.

| 0 | 1 | 2 | 3 | 4 | 5 | 6 | 7 | 8 | 9 | 10 |
| --- | --- | --- | --- | --- | --- | --- | --- | --- | --- | --- |
| No Cohesion, at all |  |  |  |  |  |  |  |  |  | Complete Cohesion |

* 17. Please write two things that helped you to get your level of Team Cohesion:

Insert Comment

* 18. Please suggest two things that would have improved your Team Cohesion:

Insert Comment

* 19. Subsection II- Engagement with the Team Based Learning (TBL) part of the clerkship:

We would like to know what you think about this TBL course as a whole, so that we can fine-tune and improve it for future classes.

Please select the rate that seems most appropriate.

| 0 | 1 | 2 | 3 | 4 | 5 | 6 | 7 | 8 | 9 | 10 |
| --- | --- | --- | --- | --- | --- | --- | --- | --- | --- | --- |
| Completely Not Engaging |  |  |  |  |  |  |  |  |  | Completely Engaging |

* 20. Please give two specific things that made this TBL course engaging for you:

Insert Comment

* 21. Please suggest two specific things that would make this TBL course more engaging:

Insert Comment

Part III- Views on the Family Medicine Discipline:

Please suggest two things that would have improved your Team Cohesion:

* 22. Changes in Perception:

We would like to know the extent to which the Family Medicine Rotation (Team Based Learning and Clerkship) affected your view of Family Medicine, as a discipline.

Please select the rate that seems most appropriate.

| 0 | 1 | 2 | 3 | 4 | 5 | 6 | 7 | 8 | 9 | 10 |
| --- | --- | --- | --- | --- | --- | --- | --- | --- | --- | --- |
| Not at all |  |  |  |  |  |  |  |  |  | To the furthest extent |

* 23. In what way(s) did the Family Medicine Rotation (Team Based Learning and Clerkship) affect your view of Family Medicine, as a discipline.

Please elaborate:

Insert Comment
