# Supplementary material for: Using team-based learning to optimize undergraduate family medicine clerkship training: mixed methods study
Source: BMC Med Educ. 2023 Jun 8;23:422. doi: 10.1186/s12909-023-04240-1 (PMC10248977; doi:10.1186/s12909-023-04240-1)
Supplement: Supplementary file 2 — Additional file 2: Appendix 2. Detailed Description of Data Collection Tool. [file 12909_2023_4240_MOESM2_ESM.docx]

# Appendix 2: Detailed Description of Data Collection Tool

The data was collected using a survey that aimed at evaluating the extent of satisfaction with the TBL in FM experience, and perceived team cohesion and level of engagement with the course throughout the experience, as well as the change in the students’ impression towards the discipline of FM. The survey was composed of three parts.

The first part was composed of five subsections, one corresponding to each of the four phases of the TBL, namely: Pre-reading, Individual test, Team test, and Team discussion during the application exercise, along with one subsection to capture the overall students’ satisfaction with the TBL in FM experience. In each of the subsections, there is a quantitative question that asked the students to rate their experience in relation to the corresponding component of the experience (i.e., each of the four phases and the overall satisfaction) on a 0 to 10 scale (0: Waste of time-10: Superb), along with the following two qualitative questions:

- Please give two reasons for your score.
- Please suggest two possible improvements to the corresponding component.

The second part of the survey was composed of two subsections, one corresponding to each of the team cohesion and the level of engagement with the course. In each subsection, the student was required to rate the extent of experiencing the respective aspect on a 0 to 10 scale (0: No cohesion, at all-10: Complete cohesion, and 0: Completely not engaging-10: Completely engaging, respectively). Besides the quantitative questions, the participating students were expected to feedback on their experience qualitatively for each aspect (i.e., team cohesion and level of engagement) as follows:

- Please write two things *that helped you to get your level of Team Cohesion*/ *that made this TBL course engaging for you*.
- Please suggest two things *that would have improved your Team Cohesion*/ *that would make this TBL course more engaging*.

The third part of the survey captured the student views/ impressions on FM with two questions; the first one is quantitative, asking the students to rate the change of their impression of the FM discipline on a 0 to 10 scale (0: Not at all & 10: To the furthest extent). The second question was qualitative, inquiring about the ways by which the respective learning experience (which entails the interplay between the in-class and clinical exposure on placements TBL in FM components) affected the impressions of the students in relation to the FM discipline.
