# Supplementary material for: Using team-based learning to optimize undergraduate family medicine clerkship training: mixed methods study
Source: BMC Med Educ. 2023 Jun 8;23:422. doi: 10.1186/s12909-023-04240-1 (PMC10248977; doi:10.1186/s12909-023-04240-1)
Supplement: Supplementary file 3 — Additional file 3: Appendix 3. Narrative Reporting on Output of Qualitative Data around Students’ Perception of TBL in FM. [file 12909_2023_4240_MOESM3_ESM.docx]

# Appendix 3: Narrative Reporting on Output of Qualitative Data around Students’ Perception of TBL in FM

The output of analysis of the qualitative data related to the TBL phases was fitted into three interrelated themes, namely: positive attributes, added value, and suggested improvements.

#### Theme 1: Positive Attributes

This theme encapsulated the segments of the qualitative data where the students identified and reflected upon aspects of the TBL in FM experience that worked to their advantage and enabled them to thrive.

The students perceived the experience to be valuable and informative in all four phases.

1-1: “...the discussion phase allowed me to stay up-to-date on the topics we need to learn about...”

2-16 “...good sources to obtain general, up-to-date knowledge on important topics in FM...”

3-20: “...the discussion has opened my eyes to concepts I would not have considered before...”

They considered the learning experience to be comprehensive and appreciated the mix of content.

1-8: “...in the clinic, you might not always come across some critical cases like asthma, Chronic Obstructive Pulmonary Disease, Transient Ischemic Attack, and many more... TBL in FM is the best way to ensure that we cover all possible cases even if we have not seen them in clinics...”

They also valued the technicalities of the TBL method, and its highlights in the learning and teaching.

1-7: “...the IRAT effectively integrated basic sciences and real-life scenarios...”

They appreciated the presence and engagement of competent, well-informed Adjunct Faculty as facilitators.

2-17 “...the facilitators always encouraged further discussions with relevant questions that are not too leading; this maximized the learning for us...”

3-25 “...different doctors from varying backgrounds, and of differing specialties, all shared with us their expertise across differing subjects within FM...”

Some students mentioned that the course appeared well organized with the right amount of material.

2-09: “...the individual test, at the end of each week, was comprehensive, and enabled us to know our areas of strengths and those that require improvement...”

The experience appeared to the students to be enjoyable and fun.

1-3: “...it is quite fun to bounce ideas off of one another...”

2-10: “...fun method of scratch cards...”

3-19: “...love the spirit and teamwork...”

The RAP (i.e., IRAT and TRAT) and Application Exercises were seen by the students as highly relevant to and applicable in the practice of FM. In addition, the students felt that the way the TBL was structured enabled them to maximize from the experience, and to apply what they were acquiring in their clinical clerkship, that was concurrently taking place.

1-08: “...very interactive! I have learned a lot of things that textbooks and clinics will not teach. It also exposed us to valuable tools and taught us how to access and use them. For example, websites that can help you get around things, and tools to assess different conditions and guidelines...”

2-14: “…spreading-out the TBLs, across the weeks, is a very good way of getting us to do them on time, instead of leaving them to the last minute... this enabled us to apply the knowledge that we were acquiring in the clinic…”

Students, who commented on the TRAT and Application exercise phases of the TBL, reported on novel, differentiating aspects:

4-34: “...this is the only rotation where I felt I was an active participant in my learning journey, where I was evaluating my own progress throughout the rotation...”

Theme 2: Added Value

This theme included the segments of the qualitative data that highlighted the immediate benefits, as well as the foreseen long-term outcome and impact of the learning experience, from the perspective of the students.

There appeared to be a consensus among the students that the TBL in FM experience encouraged them to better understand the value of the FM discipline and to develop a more positive impression of it.

1-2: “...it helped me understand how important FM doctors are and how they act as gatekeepers for the entire healthcare system...”

3-21: “...it made me appreciate FM physicians more, and their essential role in patient education, and in dealing with social and lifestyle aspects of health care...”

3-27: “...the experience of TBL in FM allowed me to develop a better understanding of FM as a discipline and as a career trajectory. Before this rotation, I would not have considered FM as a career choice...”

4-30: “...I used to consider FM as ‘boring’. This rotation shifted matters for me; I had plenty of misconceptions around the discipline and its value in the grand scheme of things...”

The students also agreed that the way the rotation was set-up fosters self-directed learning.

2-11: “...it pushed me to study every single week...”

3-22: “...I really enjoyed the group test part and the discussion within my team; this allowed me to realize my mistakes and to clarify any confusion and/ or misunderstandings in my knowledge base...”

4-36: “...the application cases were detailed and allowed for worthwhile discussions, all which really maximized the learning for me...”

From the students’ perspective, the experience enabled developing in-depth knowledge.

3-20: “...I am learning in a way deeper manner relative to how the experience would have been for me in a lecture setting...”

4-28: “...the pre-reading cases fully covered the learning objectives for the TBL and helped me build foundational knowledge on the topic...”

It also supported the students in reinforcing their existing knowledge base, and in assessing their understanding of key concepts around the subject matter.

1-7: “...the cases were effectively integrated. They taught us new information. Also, the corresponding questions solicited critical thinking, and were designed in a way to help us recall information and reinforce our understanding about the subject matter...”

The students particularly valued the emphasis on learning as opposed to performance and grading. The purpose of structuring the assessment to be ‘for learning’ and the benefit of structuring it as such was apparent to the students.

1-6: “...the IRAT tests my own knowledge and since this is not a huge part of my final score, it reduces the stress; I am able to learn freely without worrying about my GPA...”

1-8: “...in some instances, I felt that I did not score as much as I thought I would, but I was not very upset because I felt I was learning from my mistakes rather than just being punished for not scoring well...”

According to the students, the structure of the experience fostered teamwork and knowledge sharing.

2-12: “...it is nice to share ideas and brainstorm with colleagues, as well as learn to accept the other perspective even if we do not agree with it...”

5-42: “...it is always great to hear other students’ opinions. It really helps me in remembering concepts...”

The students reflected on the importance of developing a grander perspective, and a holistic, integrated approach to clinical practice, be it in FM or otherwise:

1-5: “...it helped me see more than my own point-of-view…”

4-33: “...cases revolved around multiple aspects of FM, allowing us not only to focus on the disease but on the patient, as a whole...”

Through this experience, the students developed the capacity to better understand patients’ perspectives and individual needs.

1-5: “...the TBL in FM experience grounded me in reality; it gravitated me to work towards addressing patients’ best interests...”

The experience significantly empowered the students and helped in building their resilience.

3-25: “...the randomization of the groups taught us to adapt...”

4-34: “...I really became aware of how to break down a complex and multi-factorial presentation into a list of problems and to tackle each problem independently. It has benefited my clinical reasoning...”

4-35: “...the cases kept getting more challenging as time passed, yet we were still able to handle them, as a group, which means we became more skillful...”

#### Theme 3: Suggested Improvements

This theme brought together the segments of the qualitative data which highlight opportunities for improvement and practical suggestions of how these opportunities can be capitalized upon.

Some students alluded to the heaviness of some of the content and to ways through which that can be lightened.

2-15: “...for the pre-reading materials to be briefer...”

3-27: “...the sessions go on for four hours.... some sessions feel lengthy and tiresome...”

Students mentioned ideas which they believe could further reinforce their learning and participation, including some focus on incorporating rest breaks, and on final exam assessment preparation.

1-3: “...we needed a break before the discussion starts...”

3-21 “...a bigger classroom would have allowed for more room for discussion and creativity...”

3-22: “...one group was constantly faster than the other, it would have been nice to have some pacing from the facilitators...”

Some students wanted more help from the facilitators to enhance specific aspects of the discussion.

3-25: “...more emphasis is needed on discussion about differentials and investigations...”

Some students pinpointed practical suggestions that can be incorporated as is.

3-24: “...maybe incorporate some videos...”

Ideas on how to support and further develop the students to enhance the quality of their engagement and in turn the learning experience for everyone were also brought-up.

4-36: “...a small talk at the beginning on how to give constructive feedback to colleagues would have helped...”

The students would have liked to have measures in place to ensure equal participation in the TRAT and Application cases.

2-17: “...the application case should require equal participation from all members...”

The novelty, integral to the TBL, was not unexpectedly foreign to some students. Hence, some exemplars shed light on the opportunity to support students to increase their tolerance for uncertainty and to manage their expectations around the required level of transparency within any one group.

4-37: “...I did not like having my wrong answers revealed to my group during post-test discussion...”

Some highlighted that they would want the questions across the TBL in FM rounds to be varied for fairness purposes. They appeared concerned that groups following theirs may be at an advantage relative to them due to questions leakage.

3-23: “...change the questions (in between rounds) ...”

Some students reflected upon their preferences when it comes to assessment types and questions. A few students came up with ideas to maximize their learning and for the assessment to tap into the depth of their knowledge. Questions that are more analytical in nature were called for.

3-24: “...have fewer recall questions and more analytical questions...”

4-30: “...maybe add some more very short answer questions...”

The students also called for standardization of assessments.

4-37: “...keep timing constant for each assessment...”
